# Restorative Justice Practices as a Foundation for Medical Education Innovation

**DOI:** 10.1111/tct.13852

**Published:** 2024-12-22

**Authors:** Christopher L. Klasson, Amal Shibli‐Rahhal, Eric Ortiz, Ashrita Raghuram, Jay Behel, Ada Gregory, Carrie Bernat, Nicole del Castillo

**Affiliations:** ^1^ Carver College of Medicine University of Iowa Iowa City Iowa USA; ^2^ Department of Internal Medicine University of Iowa Carver College of Medicine Iowa City Iowa USA; ^3^ Division of Behavioral Sciences RUSH Medical College Chicago Illinois USA; ^4^ Kenan Institute for Ethics Duke University Durham North Carolina USA; ^5^ Department of Psychiatry University of Iowa Carver College of Medicine Iowa City Iowa USA; ^6^ Department of Clinical Sciences Carle Illinois College of Medicine Urbana Illinois USA

**Keywords:** Burnout, Communication, Professional identity formation, Restorative justice

## Abstract

**Background:**

Restorative justice (RJ) is an ethical lens that places emphasis on a community's connection and proliferative impact of actions, promoting communication and establishing methods for accountability. RJ practices can be applied on a spectrum, including proactive community‐building practices, community discussions in response to an event, and restorative conferences addressing specific incidences of harm. This article describes an intervention that utilized RJ community‐building practices within a medical education environment and evaluates its acceptability and feasibility.

**Approach:**

During the summer of 2023, RJ interventions were planned, executed and assessed within two programmes involving pre–matriculant medical students, physician–assistant studies students and undergraduate students. The interventions utilized community‐building circles within small group activities. Capacity building included training for facilitators. Ninety‐five students participated in the RJ circles.

**Evaluation:**

Evaluation included mixed methods pre‐ and post‐intervention surveys as well as qualitative interviews. Ten students (11%) responded to the pre‐intervention survey, and 17 responded to the post‐intervention survey (18%). Seven responses were obtained from interviews and qualitative surveys. Overall, participant survey responses indicated increased feelings of connection and perceptions of mutual understanding and that the programme provided adequate space to share one's perspective. Qualitative content analysis emphasized community building through RJ circles and their ability to promote connection, meaningful vulnerability, foster peer support and a desire to continue these activities.

**Implications:**

RJ interventions within medical education environments are feasible and show considerable promise. Participants consistently noted the benefits of greater connection between peers, feelings of support and a desire for these practices to continue.

## 1 Introduction

Restorative justice (RJ) is an ethical lens and a foundational way of viewing relationships, community and conflict. It places an emphasis on the interconnectedness of individuals and the importance of perspective sharing through storytelling and addressing community members' needs collectively. RJ practices and applications include building community, resolving conflict, addressing incidents of harm and promoting healthy reintegration of community members after such harm [1]. With origins rooted in Indigenous cultures and practices [2, 3], RJ originated within the criminal justice system and is now a growing framework within academic medicine with applications including community building for students, faculty and staff; informal and formal conflict resolution; reintegration of faculty and patient safety [4, 5, 6]. Increasing calls for RJ within medicine have recognized these early efforts and invite the use of RJ in a variety of settings, including conflict resolution, patient care, and diversity and inclusion [1, 4, 5, 6, 7]. Within medical education, RJ presents a particularly attractive approach to facilitate two important phenomena—professional identity formation and resilience—through its ability to promote relational perspective sharing and learning, personal reflection and feelings of support among peers. The authors here argue that community‐building practices should be actively applied to create safe spaces within medical education for students to connect, explore important topics and react to student needs.

“Restorative justice (RJ) … places an emphasis on the interconnectedness of individuals and the importance of perspective sharing through storytelling and addressing community members' needs collectively.”

At the core of RJ are various restorative practices, ranging from communication strategies such as affective questions or statements to formal practices (i.e., community circling, conferencing, and circles of support and accountability). The practice of circling involves participants sitting in a circle with no tables or objects in the middle, a talking piece to guide a structurally equitable conversation and story‐based questions discussed in rounds. Circling is the foundation of community building and mutual understanding, which is essential to higher‐level practices such as conferencing to address conflict, harm and promote accountability. Importantly, community‐building practices exist within the spectrum of RJ practices and can be used to build connections that prevent harm proactively.

“Importantly, community‐building practices exist within the spectrum of RJ practices and can be used to build connections that prevent harm proactively.”

This paper describes two pilot programmes applying RJ community‐building practices in baccalaureate‐level and pre‐matriculant medical education environments with the goal of assessing acceptability and feasibility. Notably, the goal of these sessions was not to teach a distinct topic but rather to introduce processes that can support the overall learning environment and learners themselves with potential benefits in professional identity formation and promoting support that mitigates feelings of burnout.

“Notably, the goal of these sessions was not to teach a distinct topic but rather to introduce processes that can support the overall learning environment and learners themselves with potential benefits in professional identity formation and promoting support that mitigates feelings of burnout.”

## 2 Approach

Between 11 June 11 and 22 June 2023, we conducted two pilot programmes at the University of Iowa Carver College of Medicine that introduced RJ applications to groups of undergraduate pre‐health, medical and PA students. This study was given exempt status by the University of Iowa Institutional Review Board due to its consideration as quality assessment of an education programme.

The first pilot programme was integrated into the Summer Health Professions Education Program at the University of Iowa (SHPEP @ Iowa) in June 2023. SHPEP is a free pathway programme for 80 first‐ and second‐year undergraduate students from underrepresented backgrounds in health professions. Students receive a food stipend and stipend for completing the programme. It aims to increase students' awareness of and provide access to resources needed to pursue a career in healthcare [8]. We started with a 45‐min session during the SHPEP @ Iowa introductory meeting where we discussed community values with the participants. Four subsequent 1‐h‐long weekly circles were incorporated. Learners were assigned to the same small group throughout the programme to promote familiarity and comfort as the programme progressed.

To prepare the SHPEP small group facilitators, also called ‘circle keepers’, we administered a 2‐h training session. This training consisted of a 15‐min introduction of RJ background, participation in an hour‐long community‐building circle and a half‐hour of instruction on the procedures of leading circles. Of note, most facilitators had little to no experience with RJ practices before this programme. The circle scripts, which are the ‘roadmap’ for the circling and provide the questions that guide group discussions, were developed by the investigators and reviewed with the circle keepers for feedback. The scripts were then edited based on this feedback from circle keepers. The scripts were essential to provide consistency across small groups, especially considering the relative inexperience of the facilitators (see Appendices S1 and S2 for circle scripts for SHPEP and IMEI, respectively).

The second RJ pilot programme was integrated into the Introduction to Medical Education at Iowa (IMEI) course at the Carver College of Medicine (CCOM) at the University of Iowa. IMEI is a free 6‐week optional introductory summer programme with a stipend to cover living costs while attending that precedes the first year of medical and PA school. This programme is offered to a self‐selected group of matriculating first‐year medical and PA students who foresee a benefit from additional early preparation, such as nontraditional matriculants and nonscience majors, among others [9]. Within this setting, we organized two RJ circles in small groups that stayed together for both sessions. These circles were led by the investigator who trained the SHPEP facilitators (C.L.K.).

## 3 Evaluation

### 3.1 Quantitative Pre‐ and Post‐Surveys

Seventy‐nine students participated in SHPEP and 16 students participated in IMEI. All 95 students were invited to participate in an optional research survey via email prior and immediately after their first small group utilizing circles. Both pre‐ and post‐session surveys consisted of seven questions: five questions were adapted from the UCLA Loneliness Scale to assess disconnectedness [10], and two questions were developed by the research team to evaluate feelings of understanding and to assess the programme's ability to provide a safe space for sharing. The decision to use specific questions from the UCLA Loneliness Scale was based on the goals of the programme to build connection and actively foster support among the students. Ten students responded to the pre‐session survey (11%) and 17 responded to the post‐session survey (18%) across both programmes. The study team used the nonparametric Wilcoxon rank sum test for analysis due to the small sample size. Statistical significance was defined as a *p* value less than 0.05.Table 1 shows the participants' responses to the surveys expressed as a mean score and as the proportion of participants who chose *agree* or *strongly agree* for each survey item. Pre‐ and post‐survey results for individual items include 4.00 (SD = 0.66) to 4.12 (SD = 0.7) for *I feel ‘in tune’ with the people in my cohort*, 3.80 (SD = 0.63) to 4.12 (SD = 0.93) for *I feel like I understand my peers' backgrounds and perspectives*, 3.6 (SD = 0.84) and 3.77 (SD = 0.97) for *I feel that I have a lot in common with the people in my cohort*, 3.80 (SD = 0.78) and 4.12 (SD = 1.27) for *I feel that I have space to share my experiences with those in my cohort*, 2.10 (SD = 0.56) to 2.35 (SD = 1.58) for *I feel that my interests and ideas are not shared by those in my cohort*, 3.00 (SD = 1.05) to 2.53 (SD = 1.77) for *I feel that no one in my cohort really knows me well*, and 3.00 (SD = 1.05) to 3.82 (SD = 1.9) for *I feel that the people in my cohort really understand me*.

**TABLE 1:** Participant responses to the pre‐ and post‐session 1 survey.

Survey question	Pre‐session		Post‐session		*p* *
	Mean (SD)	Agree/Strongly Agree (%)	Mean (SD)	Agree/Strongly Agree (%)	
1. I feel ‘in tune’ with the people in my cohort	4.00 (0.66)	80	4.12 (0.7)	82	0.65
2. I feel like I understand my peers' backgrounds and perspectives	3.80 (0.63)	70	4.12 (0.93)	94	0.10
3. I feel that I have a lot in common with the people in my cohort	3.6 (0.84)	40	3.77 (0.97)	71	0.36
4. I feel that I have space to share my experiences with those in my cohort	3.80 (0.78)	60	4.12 (1.27)	88	0.12
5. I feel that my interests and ideas are not shared by those in my cohort	2.10 (0.56)	0	2.35 (1.58)	29	0.85
6. I feel that no one in my cohort really knows me well	3.00 (1.05)	30	2.53 (1.77)	35	0.30
7. I feel that the people in my cohort really understand me	3.00 (1.05)	40	3.82 (1.9)	82	**0.03**

*Note:* Answer choices ranged from 1 = *Strongly Disagree* to 5 = *Strongly Agree*. Questions 1 through 5 are from the UCLA Loneliness Scale, and questions 6 and 7 were developed by the investigators.

* *p*‐value was calculated for the mean response.

### 3.2 Qualitative Interviews and Survey Responses

Four students participated in interviews, one participant provided a narrative response to the open‐text question on the post‐session online survey, and two participants responded to the qualitative survey distributed at the end of the programme. The seven participant responses were pooled for the qualitative analysis, generating six main themes. Individual participant responses could contribute towards several themes. Responses were analysed using content analysis. No specific cut‐off for themes were set. Rather, due to the small sample size and exploratory nature of the programme, all themes expressed more than once were included. Table 2 presents the themes and representative quotes.

**TABLE 2:** Primary themes from participant responses and sample quotes.

Theme: *description* (% of coded responses)	Representative quotes
**Connection:** *Refers to feelings of connection to or with people in their cohort* (29%)	*It was really good in just establishing a camaraderie and kind of helping return humanity to us as students … because we actually see like other people have struggles. We're ripping the Band‐Aid off this social media thing where everybody's life is perfect and they are doing fine and I'm the only one who's struggling, and, so, it was kind of nice to relate and see, like, everybody had their own difficulties and challenges and how you can help someone else and then how they relate to you and vice versa on those*. *I thought it was great … it was all stuff that we would not have learned right away and it made us sort of automatically closer in a way that I do not think really anything else would have in that sense*.
**Vulnerability:** *Refers to the ability to share elements of oneself with peers* (33%)	*I feel a lot safer being open with my fellow classmates because it showed that a lot of them are … not going to judge you. They're very much willing to listen to your experiences … it was really awesome. It was one of my favourite parts of IMEI, to be honest*. *We actually did get a little bit sort of, as people got more comfortable, into, I do not want to say negatives, but like not super idealized positive … it sort of led that way into people's more realistic answers, which I thought was nice*.
**Support:** *Refers to the sense of being supported, either by peers or the process of circling* (5%)	*I think for me it is the fact that it established a precedent that moving forward in this program, we are going to continue to be open was probably the most helpful part. So like the understanding and compassion did not end in that circle. Like it continues every time we eat lunch together or anytime we are discussing topics in class. And so it was really nice to see that as the start of where in this together we are moving on this journey. We're going to stay open. We're going to stay conversant and we are going to basically be friends. So I think it had a little more lasting impact than just what happened in That circle*.
**Knowledge:** *Refers to previous knowledge of restorative justice* (5%)	*I think my knowledge base is very minimal … I have not been part of them before and I have not actually had a lot of experience with this*.
**Structure:** *Refers to participants' opinions regarding the structure and process of circling* (38%)	*The main thing was you are in a circle formation … then you are all at the same level. You're all sitting down. You're all at the same level. There's no getting up to speak. There's no nothing like that. So it kind of sets the precedence of we are in this together. We're at the same level. We're equals.’* *‘I think the biggest thing for me was … the precedent of this is an open space. We are here to share and we are going to take turns sharing and we are going to be here to support … So I think it's setting the precedent was the biggest for me and kind of feeling comfortable to proceed forward and share all the things that I had to say*.
**Application:** *Refers to suggestions for future circling applications and the willingness to participate* (19%)	*Overall, I thought it was good and can be a great benefit for future students especially when you do not know anyone*. *I would say after like major tests specifically like Step 1 or after your first week of clinicals … your big milestones, it would be nice to get in contact with that same cohort and … it would just be nice to have that as a check in*.

Overall, participants reported that the circle format contributed positively to their experience. They reported that circling fostered strong connections between peers and promoted a sense of support that carried throughout the programme. Participants reported that they would like to continue circling in the future, specifically in the context of key transitions and milestones, support groups and mentor–mentee relationships.

## 4 Implications

Despite the low response rates, our exploration suggests that establishing introductory healthcare education experiences based on a foundation of RJ is both acceptable and feasible. The survey assessing the impact of community‐building practices early in the programme's span suggested improvements in feelings of connectedness and understanding.

Themes from the qualitative analyses emphasized the sense of connectedness and support within the students' small community and the degree of trust and vulnerability that they experienced, leading to more willingness to share with authenticity.

While SHPEP facilitators were not formally assessed, anecdotal feedback was favourable toward RJ‐based programming and encouraged its continued application. The facilitators felt that they were prepared to lead the small groups, particularly with the help of the facilitator guide.

Facilitators reported that students appreciated the dedicated space for sharing their experiences and bonding with each other. They endorsed that the longitudinal application helped build rapport and their own comfort with RJ practices. Lastly, some facilitators expressed interest in further training to design and lead RJ practices independently.

Limitations include that our participant group was small and the limited number of responses obtained. We suspect that the low response rate may in part be related to recruitment methods. Specifically, email recruitment was used, and messages were addressed from study team members who the participants were not familiar with, possibly leading to those invitations being missed or ignored. Future research would benefit from establishing evaluation as a standard part of programme participation for students. A more representative sample could have expanded upon the themes discussed or provided further clarity as to what themes were most prominent. Second, the survey used was not a validated survey. To our knowledge, no validated survey exists to evaluate RJ practices, so we selected items from the validated UCLA Loneliness Scale, focusing on domains that apply to the general purpose of RJ community‐building practices. Third, data from the SHPEP and IMEI groups were combined, limiting our ability to perform distinct analyses for these different phases in healthcare education, which limits the generalizability of these results.

“In summary, our pilot programme suggests that RJ practices can be successfully introduced into medical education, particularly within small‐group teaching.”

In summary, our pilot programme suggests that RJ practices can be successfully introduced into medical education, particularly within small‐group teaching. Furthermore, our findings suggest that RJ practices can be designed to facilitate professional identity formation and help enhance resilience, both of which are important tasks in healthcare education. These programmes have served as an important foundational reference as our institution has established curriculum elements using community‐building circling for medical and PA students as well as invested further in CME‐accredited training for students, faculty and staff. This is important as a tangible example of RJ applications that can be used to answer the increasing calls for RJ implementation in medicine—an essential step at a point where most references are theoretical [7]. Future iterations of this work will include larger numbers of participants for increased analytical power and further qualitative exploration. Additionally, further work should explore implementing RJ into later stages of medical school and medical training, with potential for application in longitudinal environments to allow for innovative ways to foster support, promote self‐reflection and professional identity formation, and develop communication skills.

## Author Contributions

**Christopher L. Klasson:** conceptualization, investigation, formal analysis, project administration, writing – original draft, writing – review and editing. **Amal Shibli‐Rahhal:** formal analysis, writing – original draft, writing – review and editing. **Eric Ortiz:** writing – review and editing, formal analysis, investigation. **Ashrita Raghuram:** writing – review and editing, formal analysis. **Jay Behel:** writing – review and editing, conceptualization. **Ada Gregory:** conceptualization, writing – review and editing. **Carrie Bernat:** conceptualization, writing – review and editing. **Nicole del Castillo:** conceptualization, methodology, writing – original draft, writing – review and editing, project administration, supervision.

## Disclosure

The authors have nothing to report.

## Ethics Statement

This study protocol was evaluated by the University of Iowa Institutional Review Board and granted exempt status (IRB ID # 202305254).

## Supporting information


**Appendix S1.** Supporting Information.

**Appendix S2.** Supporting Information.

## Data Availability

The data that support the findings of this study are available from the corresponding author upon reasonable request.

## References

[1] D. Acosta and D. R. Karp , “Restorative Justice as the Rx for Mistreatment in Academic Medicine: Applications to Consider for Learners, Faculty, and Staff,” Academic Medicine 93, no. 3 (2018): 354–356. 29087964 10.1097/ACM.0000000000002037

[2] H. Zehr , The Little Book of Restorative Justice: Revised and Updated [Internet] (New York, UNITED STATES: Skyhorse Publishing Company, Incorporated, 2015), http://ebookcentral.proquest.com/lib/duke/detail.action?docID=5667816.

[3] F. E. Davis , The Little Book of Race and Restorative Justice: Black Lives, Healing, and US Social Transformation (New York NY: Good Books, 2019), https://search.ebscohost.com/login.aspx?direct=true&db=nlebk&AN=1972518&site=ehost‐live.

[4] Behel J. ‘I Shall Be Released,’ Restorative Justice Techniques Can Address Healthcare Burnout & Attrition [Internet], Reflective MedEd, 2019, retrieved February 23, 2022, https://reflectivemeded.org/2019/11/12/i‐shall‐be‐released‐restorative‐justice‐techniques‐can‐address‐healthcare‐burnout‐attrition/.

[5] A. Eniasivam , L. Pereira , and E. Dzeng , “A Call for Restorative and Transformative Justice Approaches to Anti‐Racism in Medicine,” Journal of General Internal Medicine 37, no. 10 (2022): 2335–2336. 35484366 10.1007/s11606-022-07605-2 PMC9360202

[6] I. M. Brown , “Diversity Matters: In Search of Restorative Justice,” Emerg med News. 43, no. 4 (2021): 9.

[7] G. Sawin , C. L. Klasson , S. Kaplan , et al., “Scoping Review of Restorative Justice in Academics and Medicine: A Powerful Tool for Justice Equity Diversity and Inclusion,” Health Equity 7, no. 1 (2023 Sep): 663–675. 37786530 10.1089/heq.2023.0071 PMC10541936

[8] ‘Summer Health Professions Education Program [Internet],’ retrieved July 21, 2023, Home, https://www.shpep.org/.

[9] ‘Introduction to Medical Education at Iowa|MD Program [Internet],’ retrieved July 21, 2023, https://medicine.uiowa.edu/md/student‐support/introduction‐medical‐education‐iowa.

[10] D. W. Russell , “UCLA Loneliness Scale (Version 3): Reliability, Validity, and Factor Structure,” Journal of Personality Assessment 66, no. 1 (1996): 20–40. 8576833 10.1207/s15327752jpa6601_2

