# Supplementary material for: Restorative Justice Practices as a Foundation for Medical Education Innovation
Source: Clin Teach. 2024 Dec 22;22(1):e13852. doi: 10.1111/tct.13852 (PMC11663732; doi:10.1111/tct.13852)
Supplement: Supplementary file 1 — Appendix S1. Supporting Information. [file TCT-22-e13852-s002.docx]

Supplemental Digital Content Appendix 1: Circling scripts for SHPEP program Klasson CL, et al. “Documenting the Moment: A Student Led, Mixed-Methods Pilot of Restorative Justice Practices in Medical Education Environments”

**Circle #1 – Community Guidelines**

**Welcome! We are coming together to discuss what we hope for our community while we are at SHPEP**

**Introduce talking piece**

Critical element in creating a space in which all participants can both speak and listen.

Will be passed around the circle from one person to another.

Only the person with the talking piece may speak.

Always ok to pass without answering the question.

Keeper may speak without talking piece only if it’s necessary.

Explain significance of talking piece in creating an equitable space (and personal significance if there is any).

**Check-in round**

In one sentence, describe who you are when you are at your best?

**Question 1**

Describe a time when an environment made you feel like you couldn’t be that version of yourself. What made you feel that way?

**Question 2**

Describe a time when an environment (like SHPEP) helped you feel safe, seen, and heard as that version of yourself?

**Question 3**

Now, I am going to pass out index cards and pens. Considering all that we have shared and heard today, please write down a value or community guideline that you believe would help SHPEP be a community where we can all be the best version of ourselves we described earlier.

Examples to share for reference:

- I think in our community we should listen to understand, not respond
- Speak from your own perspective (I statements, not you statements)

**Pass out note cards and pens. Let students know that they can ask for another if they have another value they would like to add. Give a minute or two for them to think or write.**

Please share the value or community guideline(s) that you wrote and describe how you believe it will benefit our community in a sentence or two.

Once you share, please place the card in the middle of the circle. We will work to compile these into a set of community values for the SHPEP program.

**Before closing the session, let the students know that for the majority of small groups at SHPEP, the group discussions will be carried out in this circling format.**

**Collect note cards after the circle is done**

**Notes for the Facilitator**

Note that in this circle there is no values/guidelines question. This is largely because of time constraint but also circle largely gets at those same questions

**Introducing the talking piece**

For the first few sessions, this will be essential for establishing how this process works. With subsequent practice, this will become less involved and intuitively understood.

**Check in Round**

Model approximately a 1-2 sentence answer (an example could be “At my best, I am a considerate friend and colleague who is passionate about creating and advocating for more equitable systems for the patients I serve”)

**Question 1**

Model a long answer (30 seconds to a minute). This should be answered in the form of a story/experience that illustrates what made you feel not supported.

**Question 2**

Model a long answer (30 seconds to a minute). This should be answered in the form of a story/experience that illustrates what made you feel not supported.

**Question 3**

Give participants a minute or two to answer. Write a value down as well and share why you believe it will be helpful.

**Circle #2 – Object Circle**

**Welcome!**

**Introduce talking piece**

Critical element in creating a space in which all participants can both speak and listen.

Will be passed around the circle from one person to another.

Only the person with the talking piece may speak.

Always ok to pass without answering the question.

Keeper may speak without talking piece only if it’s necessary.

Explain significance of talking piece in creating an equitable space (and personal significance if there is any).

**Check-in round**

What’s your name, how did you get it, and does it have a meaning that you know of?

**Values/Guidelines**

What is something that you bring to the circle today?

What is something that you need from the circle today?

Common circle guidelines:

If not mentioned, share these guidelines:

▪       Offer your full presence--no phones, try not to have your eyes wander around, etc.

▪      Listen to understand, not to respond.

▪       Shared from your own lived experience (I statements, not you statements.)

▪       Presume good intent; honor and assume impact.

▪      Acknowledge multiple truths.

▪      Confidentiality - Stories stay, lessons leave.

**Object Round**

Next we’re going to talk about the objects that we brought by explaining the story of their significance to us, and once you have shared, please put your object in the middle of the circle

**Follow Up Round**

Now pick up another person’s object, share what may have resonated with you, and return it to the original person who’s object it is

**Check-out round**

Share one word for how you’re feeling right now

**Thank you** all for being here today and for doing the circle with us

**Notes for the Facilitator**

This is the circle we went through in our training! The hope is for it to be a way to connect and learn more about the people they will all be spending time with over the next few weeks.

**Introducing the talking piece**

Nothing new to note from previous circle

**Check in Round**

Model a 2 or so sentence response.

**Values/Guidelines**

Model 1 word response

Ask each individual question as its own round. So, ask what people can bring first and pass the talking piece around, then ask what they need with another round of passing the talking piece around.

It is important to establish the common circle guidelines for the first few circles we have.

**Object Round**

Model a long answer. If you model a genuine story about your object (just like we did at the training), that is perfect.

**Question 3**

Similar to the object round, model an answer that appropriately addresses why you resonate with the object. Typically, this is shorter than the actual object round.

**Check-out round**

Model a one word answer

**Circle # 3 – Discussion about Shame**

**Introduce talking piece**

Critical element in creating a space in which all participants can both speak and listen.

Will be passed around the circle from one person to another.

Only the person with the talking piece may speak.

Always ok to pass without answering the question.

Keeper may speak without talking piece only if it’s necessary.

Explain significance of talking piece in creating an equitable space (and personal significance if there is any).

**Check-in round**

What is one word to describe how you feel right now?

**Values/Guidelines**

What is one value or attribute that you have that you believe contributes positively to the community at SHPEP? In one sentence, how do you think that value helps other people?

**Common circle guidelines:**

If not mentioned, share these guidelines:

▪       Offer your full presence--no phones, try not to have your eyes wander around, etc.

▪      Listen to understand, not to respond.

▪       Shared from your own lived experience (I statements, not you statements.)

▪       Presume good intent; honor and assume impact.

▪      Acknowledge multiple truths.

▪      Confidentiality - Stories stay, lessons leave.

**Question Round 1**

Briefly, describe what you feel shame is?

**Question Round 2**

Share a time when you have felt the emotions we just discussed in an academic environment. What about the situation or environment contributed to you feeling that way?

**Question Round 3**

Describe a time in your first part of SHPEP or another academic environment that you feel proud of – it can be either related to academics or something else. Is there anything about that story that you feel you can apply to balancing the demands of navigating pre-health curriculum and feeling empowered to do so?

**Check-out round**

Considering what we have talked about today, what is one lesson that you would like to carry forward – either here at SHPEP or in your studies in school?

**Notes for the Facilitator**

This will be on of the heavier discussions of the program. While we designed it so it doesn’t get too directly into topics, be mindful of how certain participants appear during the session and refer them if you have concerns.

**Introducing the talking piece**

**Check in Round**

Model a one word answer

**Values/Guidelines**

Answer this one to any community you feel relevant for this (which can be SHPEP if you feel you are involved to answer for)

Example answer: “I feel that I offer a sense of understanding that allows for the people around me to have grace and feel comfortable and supported to grow in areas that they want or need to”

**Question 1**

Model a 1-2 sentence response

**Question 2**

Model a long answer here. Since some facilitators are no longer in academic environments, answer to any environment you are currently in or were in the past

**Question 3**

Model a long answer here. We are trying to get at resilience strategies

**Check-out round**

Model a 1-2 sentence answer

**Circle #4 – Support at School/Workplace**

**Introduce talking piece**

Critical element in creating a space in which all participants can both speak and listen.

Will be passed around the circle from one person to another.

Only the person with the talking piece may speak.

Always ok to pass without answering the question.

Keeper may speak without talking piece only if it’s necessary.

Explain significance of talking piece in creating an equitable space (and personal significance if there is any).

**Check-in round**

In one sentence, what do you feel you contribute to the people and environments you exist in when you are at your best?

**Values/Guidelines**

What’s one value, aspect, or practice of an environment or culture that supports the best version of yourself described in the previous question?

**Common circle guidelines:**

If not mentioned, share these guidelines:

▪       Offer your full presence--no phones, try not to have your eyes wander around, etc.

▪      Listen to understand, not to respond.

▪       Shared from your own lived experience (I statements, not you statements.)

▪       Presume good intent; honor and assume impact.

▪      Acknowledge multiple truths.

▪      Confidentiality - Stories stay, lessons leave.

**Question Round 1**

Share a story of when you feel like you’ve really thrived at school, work, or an extracurricular.

**Question Round 2**

Share a story where you’ve felt undervalued and/or undermined at school, work, or an extracurricular? What happened?  How did you feel?

**Question 3**

What’s one thing that resonated with you or is lingering in your thoughts that was shared by others today?

**Closing Round**

Based on what we’ve heard, what should we do moving forward at SHPEP to better support each other?

**Notes for the Facilitator**

You may notice this is similar the previous week’s circle. However, it is written slightly differently to focus on the overall process of support and turning into a more constructive way of identifying what effective support looks like.

**Introducing the talking piece**

**Check in Round**

Model a one sentence answer

**Values/Guidelines**

Model a 2 or so sentence answer. If you can think of a story that can illustrate it or articulate why that element helps support you being your best self, that would be really useful here.

Likely don’t need the common circle guidelines at this point due to previous practice.

**Question 1**

Model a thorough answer of a story (30 seconds to a minute). Really focus on using a story here and extrapolating what allowed that situation to happen.

**Question 2**

Model a thorough answer of a story (30 seconds to a minute). Really focus on using a story here and extrapolating what allowed that situation to happen.

**Question 3**

Model 1-2 sentence answer

**Check-out round**

Model a 1-2 sentence answer

**Circle #5 – Destress/Check-in**

**Introduce talking piece**

Critical element in creating a space in which all participants can both speak and listen.

Will be passed around the circle from one person to another.

Only the person with the talking piece may speak.

Always ok to pass without answering the question.

Keeper may speak without talking piece only if it’s necessary.

Explain significance of talking piece in creating an equitable space (and personal significance if there is any).

**Check-in round**

One word to describe how you feel right now?

**Values/Guidelines**

What is something you can bring to the circle today and what is something you need from it?

**Common circle guidelines:**

If not mentioned, share these guidelines:

▪       Offer your full presence--no phones, try not to have your eyes wander around, etc

▪      Listen to understand, not to respond.

▪       Shared from your own lived experience (I statements, not you statements.)

▪       Presume good intent; honor and assume impact.

▪      Acknowledge multiple truths.

▪      Confidentiality - Stories stay, lessons leave.

**Question Round 1**

What is a small gesture/observation/bright spot that makes you happy in your daily life?

**Question Round 2**

Talk about something you are doing, either now or in the future, that you are excited about.

**Question Round 3**

In school, summer is a transition from one period to the next. Where are you now and where do you want to be.

**Closing**

One word to describe how you feel right now?

**Notes for the Facilitator**

This is meant to be a very low stress circle to touch base before the program comes to an end soon and reflect on future directions.

**Introducing the talking piece**

**Check in Round**

Model a one word answer

**Values/Guidelines**

Model a sentence answer. Since people are practiced with this type of question at this point, ask them both at one time.

**Question 1**

Model a short story (~30 seconds) of a person or thing that either regularly makes you happy or that recently happened that made you happy

**Question 2**

Model a short story (~30 seconds)

**Question 3**

Model a short story (~30 seconds). Essentially, answer to what or where you want to be in the future. This can be personal (I hope to have learned to cook this type of food), professional (I hope to have done ____), or any version of what feels authentic to you

**Check-out round**

Model a one word answer
