# Supplementary material for: Restorative Justice Practices as a Foundation for Medical Education Innovation
Source: Clin Teach. 2024 Dec 22;22(1):e13852. doi: 10.1111/tct.13852 (PMC11663732; doi:10.1111/tct.13852)
Supplement: Supplementary file 2 — Appendix S2. Supporting Information. [file TCT-22-e13852-s001.docx]

Supplemental Digital Content Appendix 2: Circling scripts for IMEI program Klasson CL, et al. “Documenting the Moment: A Student Led, Mixed-Methods Pilot of Restorative Justice Practices in Medical Education Environments”

**Before the session, ask that participants bring an object relevant to their path to medicine.**

**Welcome**

**Introduce talking piece**

Critical element in creating a space in which all participants can both speak and listen.

Will be passed around the circle from one person to another.

Only the person with the talking piece may speak.

Always ok to pass.

Keeper may speak without talking piece only if it’s necessary.

Explain significance of talking piece.

**Check-in round**

What’s your name, how did you get it, and does it have a meaning that you know of?

**Values/Guidelines (two rounds – one for bring, next for need)**

*What is something that you bring to the circle today?*

*What is something that you need from the circle today?*

Common circle guidelines:

If not mentioned, share these guidelines:

▪       Offer your full presence--no phones, try not to have your eyes wander around, etc.

▪      Listen to understand, not to respond.

▪       Shared from your own lived experience (I statements, not you statements.)

▪       Presume good intent; honor and assume impact.

▪      Acknowledge multiple truths.

▪      Confidentiality - Stories stay, lessons leave.

**Object Round**

Next we’re going to talk about the objects that we brought and then put them in the middle of the circle

**Follow Up Round**

Now pick up another person’s object and share what may have resonated with you

**Check in Question**

What brought you to IMEI?

**Final Round**

Based on what you have heard today, what is one thing you have heard from today’s circle that you would like to carry forward for the program/upcoming semester? In other words, how can we be accountable to each other?

**Check-out round**

Share one word for how you’re feeling right now

**Thank you** all for being here today and for doing the circle with us

**Introduce talking piece**

Critical element in creating a space in which all participants can both speak and listen.

Will be passed around the circle from one person to another.

Only the person with the talking piece may speak.

Always ok to pass.

Keeper may speak without talking piece only if it’s necessary.

Explain significance of talking piece.

**Opening:**

High and Low: Share your high point and low point of the program.

**Values Round**

Please share, in one or two words, the value that you need from the circle today or the value you can bring to the circle today.

Common circle guidelines:

If not mentioned, share these guidelines:

▪       Offer your full presence--no phones, try not to have your eyes wander around, etc.

▪      Listen to understand, not to respond.

▪       Shared from your own lived experience (I statements, not you statements.)

▪       Presume good intent; honor and assume impact.

▪      Acknowledge multiple truths.

▪      Confidentiality - Stories stay, lessons leave.

**Check-in round:** How does your experience with IMEI compare to what you expected coming in?

**Round 1**

When was a time that you did not feel supported before coming to IMEI? What about this experience caused you to not feel supported?

**Round 2**

When was a time you did receive that support when you needed it during the IMEI program? What was different about this from the story you told in the last question?

**Round 3**

What is something that you can take away from the past 6 weeks and the process of defining communal values that you can carry forward into future interactions/relationships at Carver?

**Check-out** **round**

Share one word for how you were feeling before this circle, and one word for how you are feeling now.
